# Do Larger Pollinators Have Higher Pollination Efficiency for the Generalized Pollination Plant *Hibiscus mutabilis*?

**DOI:** 10.3390/biology13121009

**Published:** 2024-12-04

**Authors:** Xiaoqing Shi, Bin Zheng, Xiaoli Liu, Fangwen Li, Zhangshun Zhu, Qiumei Quan, Yunxiang Li

**Affiliations:** 1 Chengdu Botanical Garden (Chengdu Institute of Park City Plant Research), Chengdu 610083, China; shixiaoqing87@126.com (X.S.); 13096355222@163.com (X.L.); 18080866159@163.com (F.L.); zzs19661104@163.com (Z.Z.); 2 College of Environmental Science and Engineering, China West Normal University, Nanchong 637009, China; zhengbin6310@163.com

**Keywords:** breeding system, *Hibiscus mutabilis*, pollen deposition, pollen limitation, pollen transfer

## Abstract

To understand the reproductive strategies of the typically introduced plant *Hibiscus mutabilis* and to compare the pollination efficiency of its different pollinators, we observed, measured, and recorded the flowering dynamics, floral traits, and visiting insects of *H. mutabilis*. Furthermore, we compared the body size, visitation rate, and pollination efficiency of the pollination insects of *H. mutabilis*. The results indicated that, despite exhibiting specialized moth pollination characteristics based on similarities in flower features to other moth-pollinated species, *H. mutabilis* actually presented a generalized pollination system. The nectar of *H. mutabilis* attracted a variety of insects to provide pollination services, a total of five flower visitors, one moth species (*Macroglossum pyrrhosticta*), and four bee species (*Xylocopa appendiculata*, *Xylocopa dissimilis*, *Bombus breviceps*, and *Apis mellifera*), were found to provide pollination services for *H. mutabilis*. The pollination efficiency of these pollinators was related to the parts of their body in contact with the stamens and stigmas of flowers. Although *M. pyrrhosticta* was larger in size and its visitation frequency in our field observations was higher, its pollination efficiency was lower. This was due to the small part of its body (proboscis) contacting the two sexual organs of *H. mutabilis* while visiting flowers, resulting in a low amount of pollen being transferred and deposited. In contrast, the bee pollinators’ proboscis was significantly shorter than that of *M. pyrrhosticta*, and it must enter the flower to suck the nectar that is hidden deeply inside the base of the flower. Therefore, the body parts of bee pollinators in contact with the two sexual organs of *H. mutabilis* were larger than *M. pyrrhosticta* in the process of visiting flowers, and the pollination efficiencies were significantly higher than those of *M. pyrrhosticta*. In addition, larger bee pollinators have higher pollination efficiency. As a result, *H. mutabilis* suffered from pollen limitation due to the pollination efficiencies of the moth pollinators in the introduced habitats, but it compensated by attracting more species of bee pollinators.

## Simple Summary

Pollinators play a crucial role in the survival and reproduction of flowering plants, and different flowering plants rely on specific types of pollinators. This study focuses on *Hibiscus* syringae and explores the effect of pollinator size on pollination efficiency. The research results indicate that although the size of pollinators is an important factor, it is not the only variable that determines pollination efficiency. In fact, the behavior patterns of pollinators and the characteristics of plant flowers themselves also have a significant impact on the reproductive process of plants. These findings provide valuable scientific reference for understanding the reproductive mechanism of hibiscus.

## 1. Introduction

Ninety percent of flowering plants are pollinated by animals [1,2,3]. Pollinators provide pollination services for plants, but they also receive pollination rewards (pollen, nectar, etc.), which is a mutually beneficial process. Without pollinators, many plants could not set seeds and reproduce, and without plants to provide pollen, nectar, and other floral rewards, many animal populations would decline [4]. Flower visitors are attracted to visit flowers by the rewards offered by plants [5,6,7], but not all flower visitors provide pollination services [8,9,10,11]. Fewer pollinator visits or less pollen delivered per visit may reduce the reproductive success of plants [12,13,14]. Therefore, floral traits may also evolve features that exclude ineffective visitors and inefficient pollinators [15]. For example, flowers of plants that rely on long-proboscis pollinators usually have long corollas to exclude other visitors; a specialized pollination relationship between pollinators and plants can maximize their mutual benefits [16,17]. 

When plants that rely on specialized pollinators for pollination are introduced into new habitats, they may suffer pollen limitation due to a shortage of pollinators [18,19] found that the pollen limitation of introduced plants was significantly different to that of native plants. Plants can improve pollination efficiency by changing floral characteristics to attract more species of visitors. The contribution of different pollinator species to a plant’s reproductive fitness differs significantly because their pollination efficiency varies greatly due to differences in the body size, foraging behavior, and visitation rate [20]. The body size of pollinators is an important functional characteristic that underlies pollination-related ecological processes [21]. The body size of pollinators such as body length and intertegular width (the distance between the bases of the left and right wings) can influence the amount of deposited pollen [22,23]. The intertegular width is a useful body size index established by Cane and Goulson found that the amount of pollen transferred by bees was influenced by the intertegular width [22,24]. Larger bees tended to deposit more pollen on stigmas at each visit than smaller bees, and the interspecific body length and intertegular width of bees had a positive effect on pollen deposition [20,23,25,26]. There was no correlation between intertegular width and pollen deposition among individuals of the same species [20]. However, whether the pollen removal and pollination efficiency (pollination efficiency = pollen deposition/pollen removal of *H. mutabilis* are affected by the body size of pollinators is still unknown [27]. 

In general, the pollination efficiency of the same type of pollinator is positively correlated with its body size [25]. However, is the pollination efficiency of different types of pollinators positively correlated with their body size? In this study, we used a generalized pollination plant *Hibiscus mutabilis* as an example to investigate the impact of pollinators’ body size on pollination efficiency. We measured the floral traits of plants and the body parameters of the various pollinator species. We identified the breeding system of *H. mutabilis* by imposing four pollination treatments. We compared the visitation rates of various pollinator species. To assess pollination efficiency, we examined the pollen deposition and pollen removal of each pollinator species. Based on field investigations, we aimed to address the following three questions: (1) What is the relationship between the pollinators’ body size and pollination efficiency? (2) What is the reproductive strategy of introduced plant *H. mutabilis*? (3) Are larger insects more efficient at pollination? (4) Are there differences in visitation rate and pollination efficiency among various pollinator species in *H. mutabilis*? (5) Is the body size of pollinators related to pollination efficiency? 

## 2. Materials and Methods

### 2.1. Study Site

**Figure 1:**
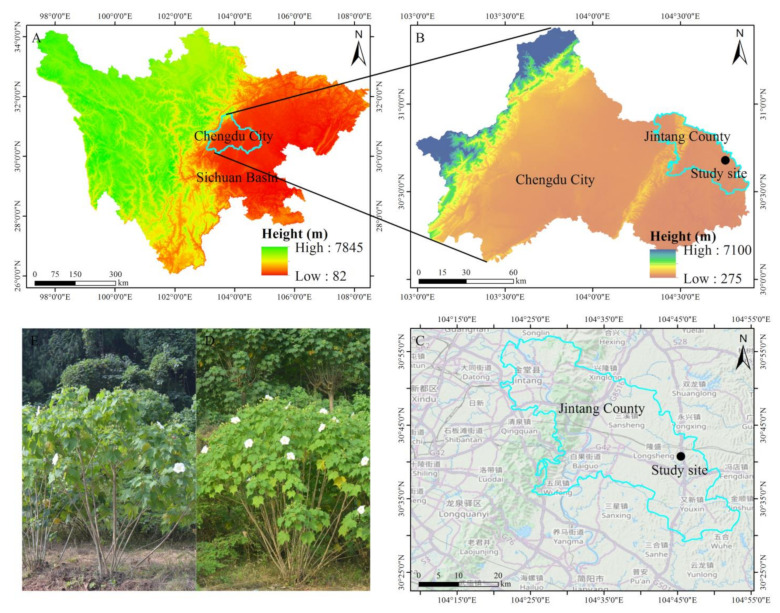
The location of the study site.

The study sites are located in Flower Valley of Zhuanlong Town, Jintang County, Sichuan Province, China (104°45′29″ E, 30°40′45″ N, 433.1 m a.s.l.) (which is located in the northeast of Chengdu Plain, and approximately 39 km away from Jintang County of Chengdu City) (Figure 1 blue circle). The study area is located in the artificial cultivation base of *H*. *mutabilis*, and approximately 200,000 *Hibiscus* plants were artificially planted. The cultivation base is surrounded by artificial secondary forests, such as *Cupressus funebris*, *Castanea mollissima*, *Eucalyptus robusta*, *Pinus species*, etc.

**Figure 2:**
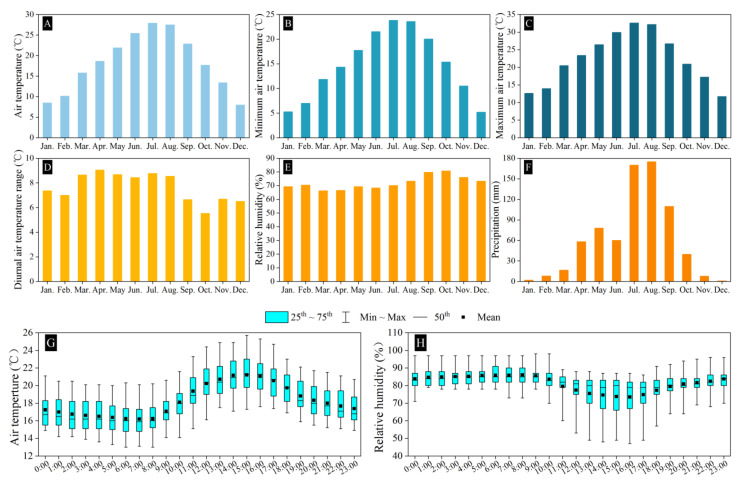
The meteorological factors of the study site from 2021 to 2023. (**A**) The monthly variations in air temperature, (**B**) the monthly minimum air temperature, (**C**) the monthly maximum air temperature, (**D**) the diurnal air temperature range, (**E**) the monthly variations in relative humidity, (**F**) the monthly precipitation, (**G**) the diurnal variation in air temperature, and (**H**) the diurnal variation in relative humidity.

This region has a subtropical monsoon climate with a moderate climate, four distinct seasons (early spring, long summer, short autumn, and winter), abundant rainfall, high humidity, cloudy skies, and a lack of sunshine. The average annual air temperature is 18.0 °C, the average annual relative humidity is 72.0%, and the precipitation is 725.7 mm. The monthly variations in the average air temperature (Figure 2A), average minimum air temperature (Figure 2B), average maximum air temperature (Figure 2C), average diurnal air temperature range (Figure 2D), average relative humidity (Figure 2E), and average precipitation (Figure 2F) of the study site from 2021 to 2023 and the diurnal variations in air temperature and relative humidity in October 2023 (the peak blooming period of *H. mutabilis*) are shown in Figure 2. These meteorological data were obtained from the Sichuan Meteorological Bureau.

### 2.2. Study Species

**Figure 3:**
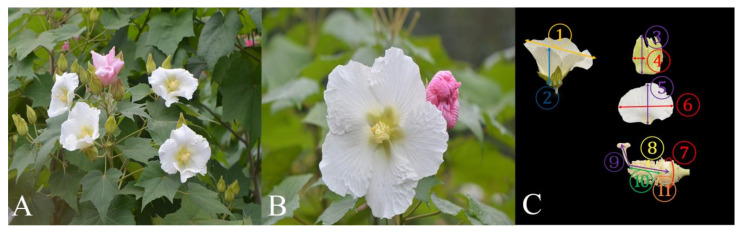
Floral traits of *Hibiscus mutabilis*. (**A**) the inflorescences, (**B**) the open flower (white flower) and the withered flower (red flower), and (**C**) floral parameters. The serial numbers in Figure 3C represent the flower parameters of *Hibiscus mutabilis*: ① the height of corolla, ② the diameter of the corolla, ③ the length of sepal, ④ the width of sepal, ⑤ the length of petal, ⑥ the width of petal, ⑦the circumference of floral base, ⑧ the length of stamen, ⑨ the length of pistil, ⑩ the length of staminal column, and ⑪ the height of ovary.

*Hibiscus mutabilis* Linnaeus (Malvaceae) is a perennial deciduous shrub or small tree 2–5 m tall with broadly ovate to round ovate or cordate leaves. *H*. *mutabilis* originated in the Hunan Province of China and was widely cultivated in many areas of both North and South China as a garden ornamental plant because of its large and beautiful flowers [28]. The flowering period of *H*. *mutabilis* usually occurs from July to November each year, while the peak blooming period occurs in October; the longevity of a single flower is only 1~2 days. The hermaphroditic and actinomorphic flowers are solitary and axillary on the upper branches (Figure 3A). Each flower consists of five petals, one staminal column, five styles, and one campanulate calyx. The corolla is white or reddish (Figure 3B). The nectary is located at the base of the calyx.

### 2.3. Floral Traits

In the peak blooming period of *H. mutabilis* in 2023, at least 30 flowers from 15 plants were randomly selected at the bud stage and dissected to measure the morphological parameters (including the corolla diameter; the height of the corolla; the length and width of the sepal; the length and width of the petal; the length of the stamen; staminal column; and the pistil; the height of the ovary; the diameter of the ovary; and the circumference of the floral base) (Figure 3C) using a digital vernier caliper with an accuracy of 0.01 mm (Syntek: Deqing Shengtaixin Electronic Technology Co., Ltd., Taiwan, China) at the first day of flowering. The circumference of the floral base was measured by a non-stretchable thread, then the length of the thread was determined by the vernier caliper. The measurement of flower parameters is shown in Figure 3C. 

### 2.4. Pollen/Ovule Ratio (P/O)

In the peak blooming period of *H. mutabilis* in 2023, at least 30 immature flowers from 15 plants were randomly selected to count the number of pollen grains and ovules and to determine the pollen/ovule (P/O) ratio. The number of ovules per flower was counted using a stereoscopic microscope (Leica S5E: Leica Microsystems AG, Wetzlar, Germany). The anthers of each flower were dissected and washed in 400 mL of distilled water to dislodge the pollen grains from the anthers. The suspensions were stirred for 2 min, and three 1 mL samples of suspension were drawn, respectively, on the plankton counting chambers, after which the number of pollen grains in the samples was counted using a binocular microscope with a light microscope at ×10 magnification (Leica DM500: Leica Microsystems Ltd., Wetzlar, Germany). The pollen count of the three sub-samples (1 mL each) was averaged and multiplied by the dilution factor (400) to obtain the total number of pollen grains per flower. 

### 2.5. Nectar Properties

To evaluate the nectar crop of *H. mutabilis* per flower, at least 30 buds from 15 plants were randomly selected, labeled, and bagged in the peak blooming period, and the volume of secreted nectar and the sugar concentration of the nectar were measured using 10 µL glass microcapillary tubes (Hirschmann Laborgerate, Eberstadt, Germany) and a hand-held refractometer (WYT: Chengdu Haochang Photoelectric Instrument Co., Ltd., Chengdu, China), respectively, at 5:00 p.m. on the first day of flowering, respectively. The refractometer readings were converted to grams of solute per 100 mL solution, and the calculation method is referenced from Bolten [29].

### 2.6. Foraging Behavior Observations

To estimate the types of floral visitors and their foraging behaviors (for pollen or/and nectar) in *H. mutabilis*, we (four observers) observed the foraging behaviors of various visitors in the period from 7:00 to 17:30 on sunny days in the peak blooming period. Four plots (2 m × 2 m) were randomly established, each including 10 flowers. These plots were observed daily and every 30 min. A total of 128 observation sessions were conducted. The pollinator types, the foraging behaviors of every visitor, the time spent visiting a flower (TSVF), and the number of flowers visited were recorded, and the visit frequency (VF) of each visitor (visits per flower per 30 min) was calculated by dividing the total number of observed flowers by the number of flowers visited per 30 min. The images of visitors were captured using a camera (Nikon DSLR, D7000, 16.2 megapixels). All flower visitors were captured and preserved in the Herbarium of China West Normal University for subsequent identification and measurements in the laboratory. The main pollinators were determined based on the number of visits and the foraging behaviors. 

### 2.7. Pollen Deposition and Pollen Removal

To compare the pollination efficiency of different pollinators, at least 100 buds from 20 plants of *H. mutabilis* were randomly selected and bagged in fine-mesh polyester bags with 0.18 mm aperture in the peak blooming period. The bags were removed after the flowers bloomed and were exposed to the visitors, and the flowers were harvested immediately after they were visited a single time. Then, the stigma was dissected and washed in 5 mL of 75% alcohol to dislodge the pollen grains from the stigma. The suspension was stirred for 2 min, and the sample of suspension was drawn on the plankton-counting chamber, after which the number of pollen grains in the sample was counted under a light microscope at ×10 magnification, that is, the amount of pollen deposition (PD) on the stigma after the flower was visited by the visitors. Moreover, we counted pollen grains remaining in the anthers of the flowers following the methods described above. Pollen removal per flower was calculated as the mean number of pollen grains per flower minus the remaining grains per flower, and the pollen removal rate (PRR) was calculated by dividing the pollen removal by the mean number of pollen grains per flower. Finally, the pollination efficiency (PE) was calculated by dividing the pollen deposition by the pollen removal.

### 2.8. Measurements of Body Size of Pollinators

To measure the body size of all pollinators, we captured 10 individual insects of each species visiting the flowers of *H. mutabilis* in the peak blooming period. The insect specimens were pinned and dried in a specimen box. The specimens were photographed using a stereoscopic microscope JSZ6S with a digital camera (Nanjing Jiangnan Yongxin Optical Co. Ltd., Nanjing, China). After imaging, the body parameters (including the length of the body (LB), the width of the unfolded wings (WUW), the length of the head (LH), the width of the head (WH), the length of the chest (LC), the width of the chest (WC), the length of the abdomen (LA), the width of the abdomen (WA), the width of the base-wing (WBW), and the length of the proboscis (LP)) were measured using ImageJ 1.8.0 software (NIH Image, Research Services Branch, Reston, WV, USA). 

### 2.9. Breeding System

To identify the breeding system of *H. mutabilis*, at least 160 buds from 30 plants were randomly selected, labeled, bagged, and subjected to four pollination treatments during the peak blooming period. (i) Open-pollination treatment (OPT): 40 buds from 30 plants were labeled and exposed to open-pollination as natural control without any other treatment. (ii) Autonomous self-pollination treatment (ASPT): 40 buds from 30 plants were labeled and bagged in fine-mesh polyester bags to exclude any insects, and no other manipulations were performed. (iii) Self-pollination treatment (SPT): 40 buds from 30 plants were labeled and bagged in fine-mesh polyester bags, and the stigmas were hand-pollinated with self-pollen grains from flowers of the same individual after the flowers opened. (iv) Cross-pollination treatment (CPT): 40 buds from 30 plants were labeled and bagged in fine-mesh polyester bags, and the stigmas were hand-pollinated with outcross pollen grains from multiple flowers of other individuals after the flowers opened. Four weeks later, the fruits produced by these flowers were hand-harvested, and the number of seeds and undeveloped ovules in each fruit was counted.

### 2.10. Statistical Analyses

Gray correlation analysis and Spearman’s correlation analysis were used to analyze the influences of body sizes (including the length of the body and proboscis, the length and width of the head, the chest, and the abdomen) of pollinators on pollination efficiency (including the time spent visiting a flower, the number of pollen deposition and the pollen removal rate). The one-way analysis of variance (ANOVA) (with Duncan’s multiple range test) was used to compare the seed set with the different pollination treatments, the pollination efficiency (PE) of different visitors (including the time spent visiting a flower (TSVF), the amount of pollen deposition (PD), and the pollen removal rate (PRR)), and the body size between the different pollinators (including the length of the body (LB), the width of the unfolded wings (WUW), the length of the head (LH), the width of the head (WH), the length of the chest (LC), the width of the chest (WC), the length of the abdomen (LA), the width of the abdomen (WA), the width of the base-wing (WBW), and the length of the proboscis (LP)). 

Origin-Pro 9.0, ArcGIS 10.2, and Photoshop software were used for data processing and graph construction. 

## 3. Results

### 3.1. Floral Traits

The hermaphroditic and actinomorphic flowers of *H. mutabilis* are single- or double-flowered on the upper branches (Figure 3A), and the floral diameter is 128.79 ± 1.54 mm (Table 1). The nearly round petals form a corolla about 10 mm deep, and the nectary is located at the base of the calyx. The petals are white at the early blooming of flowers and gradually turn red until the petals re-close (Figure 3B). Each flower has approximately 141.8 ± 2.3 stamens, which forms a staminal column around the base of the style. The pistil is composed of five stigmas. Although the forepart of the pistil curves markedly upward, its length is significantly longer than the staminal column (χ^2^ = 44.264, *n* = 60, df = 1, *p* < 0.001) and stamen (χ^2^ = 44.262, *n* = 60, df = 1, *p* < 0.001) (Table 1). 

### 3.2. Pollen/Ovule Ratio (P/O)

The numbers of pollen grains and ovules per flower of *H. mutabilis* were 72813.30 ± 1220.51 (*n* = 30) and 241.93 ± 2.80 (*n* = 30), respectively (Table 1). The P/O ratio of *H. mutabilis* was 303.22 ± 5.91 (*n* = 30), indicating that it was an obligatory outcrossing plant according to the criteria proposed by Cruden [30]. 

### 3.3. Nectar Properties

Nectar secreted by the nectary at the base of the calyx is stored at the base of the ovary, and nectar secretion occurs throughout the flowering process. Although the longevity of a single flower of *H. mutabilis* was shorter, each flower can secrete 13.26~36.10 μL of nectar with an approximately 11.47~38.98 g/100 mL sugar concentration (Table 1). 

### 3.4. Foraging Behavior of Pollinators

**Figure 4:**
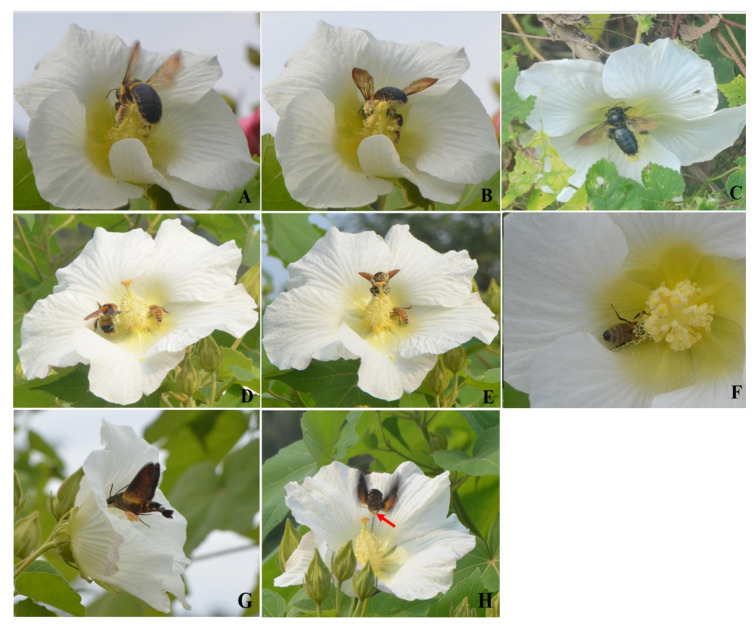
Diverse visitors visiting flowers of *Hibiscus mutabilis*: (**A**–**F**) four bee species visiting flowers ((**A**,**B**), *Xylocopa appendiculata*; (**C**) *Xylocopa dissimilis*; (**D**,**E**), *Bombus breviceps*; and (**F**) *Apis mellifera*); (**G**,**H**) one moth species visiting a flower (*Macroglossum pyrrhosticta*, the red arrow represents the pollen of *H. mutabilis*) visiting a flower.

*H. mutabilis* only opened in the daytime; the petals began to close in the evening and the longevity of the single flower was approximately 6~12 h on sunny days. On rainy days, the duration of a single flower can be prolonged from 1 to 2 days. A total of five insect species provided pollination services for *H. mutabilis* in the field observation (Figure 4), including four bee species (*Xylocopa appendiculata*, *Xylocopa dissimilis*, *Bombus breviceps*, and *Apis mellifera*) (Figure 4A–F) and one moth species (*Macroglossum pyrrhosticta*) (Figure 4G,H). In addition, some occasional visitors such as beetles and flies were found to land on the leaves or petals of *H. mutabilis*. However, they only stayed for a short time and no part of their body touched the two sexual organs of *H. mutabilis*. Therefore, they cannot be considered effective pollinators of *H. mutabilis*. The petals of *H. mutabilis* were closed at night, and the temperature at night in October was lower; hence, no visitors were observed visiting the flowers of *H. mutabilis* at night.

All pollinators visited the flowers of *H. mutabilis* for nectar and did not actively collect the pollen grains. The bee visitors entered the corolla to find and suck the nectar stored at the base of the ovary, and when the nectar was consumed by them, they turned their body’s direction and emerged from the flower. In this process, their chest and abdomen touched the stamen group carrying a large amount of pollen grains away from the chambers and depositing some of the pollen grains on the stigmas. Thus, the whole pollination process is completed. In contrast, *M. pyrrhosticta* ceaselessly inflated its wings and kept its body suspended in the air or grasped the stigmas with its front legs, rested its chest on the stigmas, and put its proboscis into the flower of *H. mutabilis* through the entrance of the corolla to suck the nectar. In the process of sucking the nectar, the proboscis and the front legs of *M. pyrrhosticta* touched the stamens and stigmas of *H. mutabilis*, while its chest did not touch the anthers and carried pollen grains from it (Figure 4H).

### 3.5. Visiting Frequency

**Figure 5:**
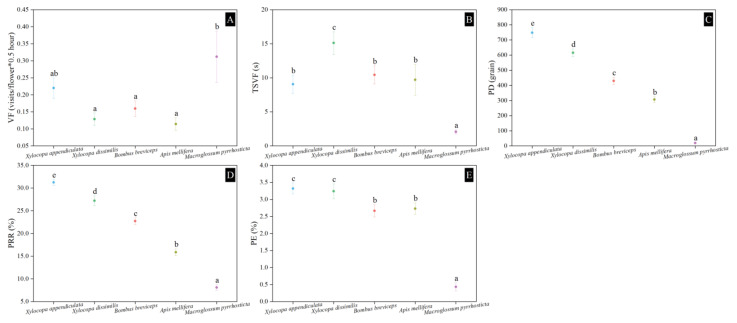
Visiting frequencies, pollen deposition, pollen removal rate, and pollination efficiencies of pollinators in *Hibiscus mutabilis.* (**A**) VF, visiting frequency; (**B**) TSVF, the time spent visiting a flower; (**C**) PD, the amount of pollen deposition per visit; (**D**) PRR, the rate of pollen removal from anthers per visit; and (**E**) PE, pollination efficiency per visit. The lowercase letters indicate statistically significant differences in the nectar standing crop at *p* < 0.05 with the one-way ANOVA test.

The visiting frequency of the moth pollinator of *M. pyrrhosticta* in *H. mutabilis* was the highest (0.31 ± 0.08 visits flower^−1^∙0.5 h^−1^), significantly higher than that of the other pollinators (*F*_4,105_ = 4.165, *p* = 0.004), followed by *X. appendiculata* (0.22 ± 0.03 visits flower^−1^∙0.5 h^−1^), *B. breviceps* (0.16 ± 0.02 visits flower^−1^∙0.5h^−1^), and *X. dissimilis* (0.13 ± 0.02 visits flower^−1^∙0.5 h^−1^). The lowest visiting frequency was for *A. mellifera* (0.11 ± 0.02 visits flower^−1^∙0.5 h^−1^) (Figure 5A), and significantly lower than that of the other three bee pollinators (*F_3_*_,84_ = 4.232, *p* = 0.008). In contrast, the time spent visiting a flower of *M. pyrrhosticta* was 2.07 ± 0.30 s, which was significantly lower than that of the other bee pollinators (*F*_4,100_ = 9.526, *p* < 0.001) (Figure 5B). The time spent visiting a flower of *X. dissimilis* was the longest amongst the bee pollinators, approximately 15.12 ± 1.66 s, followed by *B. breviceps* (10.4*2* ± 1.33 s) and *A. mellifera* (9.70 ± 2.26), and the shortest time was for *X. appendiculata*, only 9.05 ± 1.33. There were no significant differences in the time spent visiting a flower amongst the four bee pollinators (*F*_4,80_ = 2.658, *p* = 0.054). 

### 3.6. Pollen Deposition and Removal

*M. pyrrhosticta* deposited approximately 19.40 ± 3.49 of pollen grains on the stigma (Figure 5C) and took away approximately 8.10% ± 0.63% of pollen grains from the stamen group (Figure 5D) of *H. mutabilis* per visit; these were significantly lower than the bee visitors (PD: *F_4_*_,100_ = 150.346, *p* < 0.001; PRR: *F_4_*_,100_ = 141.850, *p* < 0.001). Similarly, the pollination efficiency of *M. pyrrhosticta* was lower (0.43% ± 0.12%), and significantly lower than that of bee pollinators (*F_4_*_,100_ = 46.725, *p* < 0.001) (Figure 5E). The pollen deposition (748.3 ± 32.9), pollen removal rate (31.24% ± 0.63%), and pollination efficiency (3.32 ± 0.16) of *X. appendiculata* were the highest among the four species of bee pollinators, and significantly higher than those of the other three bee pollinators (PD: *F_3_*_,80_ = 58.061, *p* < 0.001; PRR: *F_3_*_,80_ = 67.058, *p* < 0.001; PE: *F_3_*_,80_ = 3.381, *p* = 0.022).

### 3.7. The Body Size of Pollinators

**Figure 6:**
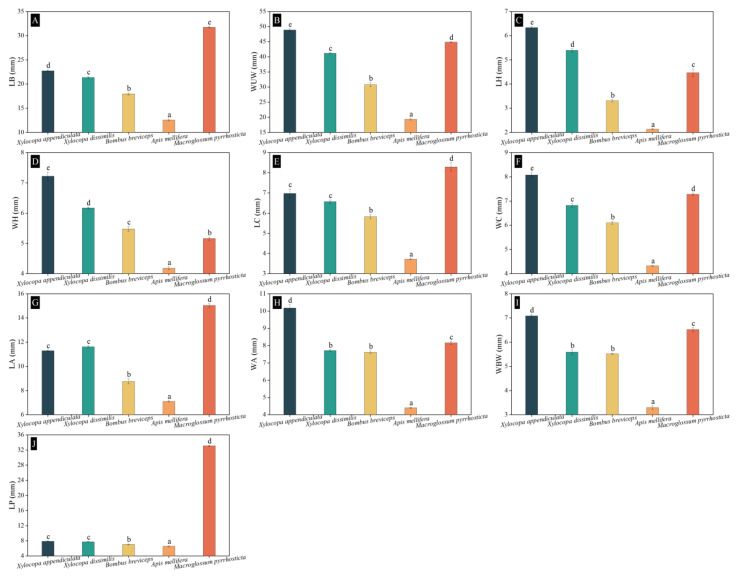
The body parameters of pollinators of *Hibiscus mutabilis*. (**A**) LB, the length of the body; (**B**) WUW, the width of the unfolded wings; (**C**) LH, the length of the head; (**D**) WH, the width of the head; (**E**) LC, the length of the chest; (**F**) WC, the width of the chest; (**G**) LA, the length of the abdomen; (**H**) WA, the width of the abdomen; (**I**) WBW, the width of the base-wing; and (**J**) LP, the length of the proboscis. The lowercase letters indicate statistically significant differences in the nectar standing crop at *p* < 0.05 with the one-way ANOVA test.

The body parameters of pollinators of *H. mutabilis* are shown in Figure 6. The body size of *M. pyrrhosticta* is the largest of all pollinators of *H. mutabilis*, and its LB (31.72 ± 0.18 mm) (Figure 6A), LC (8.29 ± 0.21 mm) (Figure 6E), LA (15.04 ± 0.14 mm) (Figure 6G), and LP (33.11 ± 0.14 mm) (Figure 6J) were significantly larger than those of the other four bee pollinators of *H. mutabilis* (LB: *F_4_*_,50_ = 1184.745, *p* < 0.001; LC: *F_4_*_,50_ = 126.301, *p* < 0.001; LA: *F_4_*_,50_ = 547.686, *p* = 0.022; LP: *F_4_*_,50_ = 82181.181, *p* < 0.022). The body size of *X. appendiculata* was the largest among the four species of bee pollinators of *H. mutabilis*, followed by *X. dissimilis* and *B. breviceps*, and *A. mellifera* was the smallest. And there were significant differences in body sizes between the bee pollinators of *H. mutabilis* (*p* < 0.001).

### 3.8. Relationships Between Pollen Transfer and Body Size of Pollinators

**Figure 7:**
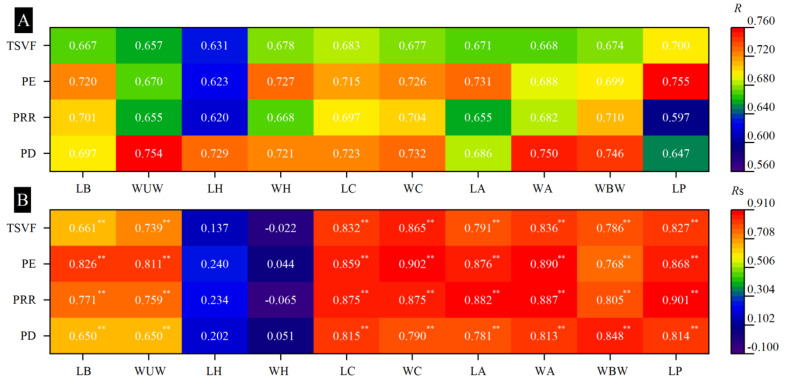
The effects of the body parameters of bee pollinators (including the length of the body (LB), the width of the unfolded wings (WUW), the length of the head (LH), the width of the head (WH), the length of the chest (LC), the width of the chest (WC), the length of the abdomen (LA), the width of the abdomen (WA), the width of the base-wing (WBW), and the length of the proboscis (LP) on the pollen deposition (PD), pollen removal rate (PRR), pollination efficiency (PE), and time spent visiting a flower (TSVF) were analyzed via gray correlation analysis and Spearman’s correlation analysis. (**A**) the gray correlating degree, (**B**) Spearman’s correlation coefficient. ** indicated that Spearman’s correlation is significant at 0.01 levels.

The correlation analysis results between the body parameters of bee pollinators and their pollination efficiency are shown in Figure 7. The results of the gray correlation degree analysis showed that the body parameters of bee pollinators had higher contributions to pollination efficiency (*R* > 0.55) (Figure 7A). Except for the length and width of the head of bee pollinators, which showed a weak correlation with pollen deposition, pollen removal rate, pollination efficiency (*p* > 0.05), and time spent visiting a flower, the other body parameters (including the length of body (LB), the width of unfolded wings (WUW), the length of the chest (LC), the width of the chest (WC), the length of the abdomen (LA), the width of the abdomen (WA), the width of the base-wing (WBW), and the length of the proboscis (LP)) showed significant positive correlations with pollination efficiency (*p* < 0.01) (Figure 7B).

**Figure 8:**
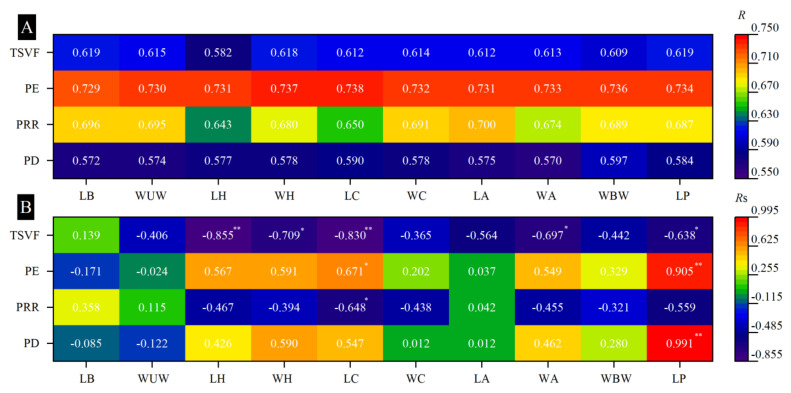
The effects of the body parameters of *Macroglossum pyrrhosticta* (including the length of the body (LB), the width of the unfolded wings (WUW), the length of the head (LH), the width of the head (WH), the length of the chest (LC), the width of the chest (WC), the length of the abdomen (LA), the width of the abdomen (WA), the width of the base-wing (WBW), and the length of the proboscis (LP)) on the pollen deposition (PD), pollen removal rate (PRR), pollination efficiency (PE), and time spent visiting a flower (TSVF) were analyzed via gray correlation analysis and Spearman’s correlation analysis. (**A**) The gray correlating degree; (**B**) Spearman’s correlation coefficient. * and ** indicate that Spearman’s correlation is significant at 0.05 and 0.01 levels, respectively.

Like bee pollinators, the body size of *M. pyrrhosticta* had a greater impact on pollination efficiency (*R* > 0.55) (Figure 8A). The Spearman correlation analysis showed that the length of the proboscis of *M. pyrrhosticta* was positively correlated with the pollen deposition (*R*s = 0.991, *p* < 0.001); the length of the proboscis (*R*s = 0.905, *p* < 0.001) and the length of the chest (*R*s = 0.671, *p* = 0.034) were positively correlated with the pollination efficiency; the length of the chest was negatively correlated with the pollen removal rate (*R*s = −0.648, *p* = 0.043); and the length of the head (*R*s = −0.855, *p* = 0.002), the width of the head (*R*s = −0.709, *p* = 0.022), the length of the chest (*R*s = −0.830, *p* = 0.003), the width of the abdomen (*R*s = −0.697, *p* = 0.025), and the length of the proboscis (*R*s = −0.638, *p* = 0.047) were negatively correlated with the time spent visiting a flower.

### 3.9. Breeding System

**Figure 9:**
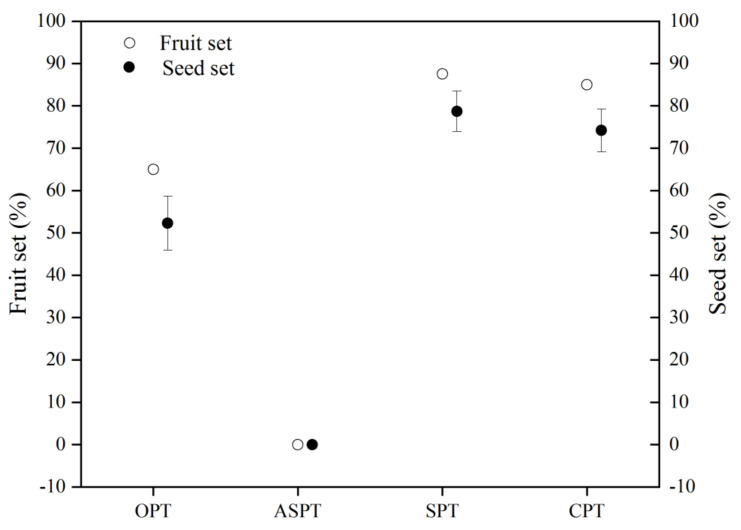
Pollination treatments of *Hibiscus mutabilis*. OPT open-pollination treatment; ASPT, autonomous self-pollination treatment; SPT, self-pollination treatment; CPT, cross-pollination treatment.

The fruit set and the seed set of *H. mutabilis* are shown in Figure 9. Approximately 65.0% of *H. mutabilis* flowers bore the fruit under natural conditions; the flowers treated with hand self-pollination (87.5%) and hand cross-pollination (85.0%) would produce the fertile seeds, and the flowers that were bagged failed to bear fertile seeds (Figure 9). This indicates that *H. mutabilis* is highly dependent on pollinators to provide pollination services to produce fruit. *H. mutabilis* could produce 52.28% ± 6.37% fertile seeds in natural conditions, which was significantly lower than the seed sets from hand self-pollination (78.72% ± 4.82%) (χ^2^ = 15.699, *n* = 80, df = 1, *p* < 0.001) and hand cross-pollination (74.18% ± 5.06%) (χ^2^ = 7.507, *n* = 80, df = 1, *p* = 0.006) (Figure 9). This suggested that there was a pollen limitation under natural conditions. 

## 4. Discussion and Conclusions

Floral traits are a combination of characteristics that plants exhibit to attract pollinators. They are formed in the process of long-term adaptation to their pollinators, and they reflect their adaptation to a certain kind of pollinators [31,32,33]. The flowers of *H. mutabilis* with only bagged treatment in the field treatments failed to bear the fertile seeds, and the P/O ratio was as high as 303.22 ± 5.91, indicating that the breeding system of *H. mutabilis* is prone to cross-pollination and its pollination process requires pollinators. In fact, the moth *M. pyrrhosticta* was observed to provide pollination services to *H. mutabilis* in our field observation; meanwhile, approximately 24.61 ± 1.08 μL of nectar per flower was secreted by *H. mutabilis* and provided to *M. pyrrhosticta* as the pollination rewards. However, the nectary of *H. mutabilis* is located at the base of the calyx, and the nectar secreted by the nectaries is stored at the base of the ovary, and the long flower corolla can prevent some visitors from easily sucking the nectar, which is similar to the floral characteristic displayed by other plants (such as *Angraecum arachnites* (Orchidaceae) [34], *Cynorkis uniflora* (Orchidaceae) [35]; *Clarkia concinna* and *Clarkia breweri* (Onagraceae) [36]; *Lagenaria siceraria* (Molina) Standl. (Cucurbitaceae), *Luffa acutangula* (L.) Roxb. (Cucurbitaceae), *Trichosanthes anguina* L. (Cucurbitaceae), and *Trichosanthes kirilowii* Maxim (Cucurbitaceae) [37]; *Linanthus dichotomus* (Polemoniaceae) [38]; *Bonatea antennifera* (Orchidaceae) [39] that rely on moth pollination. The moth *M. pyrrhosticta* has a long proboscis (33.11 ± 0.14 mm), the length of which is highly consistent with the length of floral corolla (30.97 ± 1.15 mm) of *H. mutabilis* (χ^2^ = 1.601, n = 40, df = 1, *p* = 0.206), and it does not need to enter the flower to suck the nectar stored at the base of the ovary and only its proboscis and front legs can touch the stigmas and stamens of *H. mutabilis* in the process of visiting flowers. Tanaka [40] found that the introduced plant of *Hibiscus syriacus* L. in Japan, a plant native to China with flower characteristics similar to *H. mutabilis*, was highly dependent on the hawk moth (*Cephonodes hylas*) for pollination. Therefore, *H. mutabilis* is mainly dependent on specialized pollinators-moth species for pollination, although no reports have been published about the moth pollination for *H. mutabilis*.

Herkogamy is one of the effective mechanisms to avoid self-pollination in flowering plants [11,16,41]. The up-curved stigma group was significantly higher than the staminal column (*p* < 0.001), which can effectively reduce the probability of self-pollination when *M. pyrrhosticta* visits flowers. However, the ability to carry pollen was limited due to the small body part (the proboscis and front legs) of *M. pyrrhosticta* in contact with the two sexual organs of *H. mutabilis* in the process of visiting flowers. Only approximately 19.4 ± 3.5 of pollen grains were deposited on the stigmas of *H. mutabilis* and approximately 0.75% ± 0.63% of pollen grains were carried away from the anthers of *H. mutabilis* in a process of visiting flowers of *M. pyrrhosticta*, and the number of pollen deposition on stigma was significantly lower than the number of the ovule of *H. mutabilis* (χ^2^ = 35.323, *n* = 50, df = 1, *p* < 0.001). Therefore, *H. mutabilis* may suffer from the shortages of cross-flower pollen.

Although *H. mutabilis* did not have the ability of automatic self-pollination and only a few pollen grains were deposited on the stigma of *H. mutabilis* after a visit to the flower by *M. pyrrhosticta*, it can produce 52.28% ± 6.37% fertile seeds under natural conditions, indicating that *H. mutabilis* did not suffer from severely pollen limitation. Previous studies have shown that the introduced (or invasive) plants are more susceptible to pollen limitation [42,43,44], plants can reduce this pollen limitation by changing the floral traits (such as reward quality, population density, and enemy escape) to attract more pollinators [19,45,46] or the breeding system (such as self-compatibility and autonomously self-pollination) [47]. Hence, the invasive (or introduced) species do not suffer more pollen limitation than the natives [19,44]. *H. mutabilis* has five nectaries and five petals; the nectaries are located in the base between the two petals, and the nectar secreted by the nectaries is hidden in the gap between the bases of the two petals, so the flower structure of *H. mutabilis* shields visitors with a shorter proboscis. The peak blooming period of *H. mutabilis* occurs in October; the air temperature during the study period was approximately 16.2–21.2 °C (Figure 2G), and only *Pharbitis limbata* (Convolvulaceae) and a few species of Compositae were flowering in the habitat. Therefore, the food sources for bee pollinators are not sufficient. Hence, the pollination rewards offered by *H. mutabilis* to *M. pyrrhosticta* are attractive to generalized bee pollinator species. In fact, except for *M. pyrrhosticta*, four bee species (*X. appendiculata*, *X. dissimilis*, *B. breviceps*, and *A. mellifera*) were observed to provide pollination services for *H. mutabilis* in the field observations. 

The length of the proboscis of bee pollinators is shorter than the length of the floral corolla of *H. mutabilis* (χ^2^ = 50.706, *n* = 70, df = 1, *p <* 0.001), so the bees must enter the flowers and suck the nectar stored at the base of the ovary. After the bee visitors consumed the nectar secreted by one nectary, they turned their bodies to continue sucking the other nectar until all the nectar in the flower had been consumed. The pollen grains of *H. mutabilis* are very sticky and stick to the body surface of the pollinators (such as the proboscis, legs, chest, and abdomen) and are transferred to the stigmas. A large number of pollen grains from the same flower of *H. mutabilis* were transferred to the stigma by the bee pollinators so that it was no longer affected by the pollen limitation. In fact, *H. mutabilis* produced 78.72% ± 4.82% of fertile seeds via the hand self-pollination treatment (Figure 9).

The flowers of *H. mutabilis* had a generalized pollination system and attracted various pollinator species by providing plenty of nectar rewards. Because of the differences in the pollination parts of the body between moth and bee pollinators, their body size has different effects on pollination efficiency. Although the length of the proboscis of pollinations could significantly affect the amount of pollen deposition (*p* < 0.001), *M. pyrrhosticta* relies on its proboscis to provide pollination services for *H. mutabilis,* and other parts of the body have little effect on pollination efficiency. *M. pyrrhosticta,* with a longer proboscis, was able to deposit more pollen grains on the stigma of *H. mutabilis* and was also able to consume the nectar stored at the base of the flower more quickly. In contrast, bee pollinators mainly rely on other parts of their body (such as the proboscis, legs, chest, and abdomen) to provide pollination services for *H. mutabilis*, so the larger bee pollinators are able to transfer and deposit more pollen grains. The results showed that the larger body size of bee species *X. appendiculata* had the highest pollen deposition, pollen removal rate, and pollination efficiency, followed by *X. dissimilis* and *B. breviceps*; the body size of *A. mellifera* is the smallest, and its pollen deposition, pollen removal rate, and pollination efficiency were the lowest. The pollen grains of *H. mutabilis* are very sticky, and pollen grains can adhere to bee pollinators with a larger body compared to smaller bee pollinators. In addition, bee pollinators with a larger body size have less flexibility to rotate their bodies in flowers than smaller bee pollinators, and they spend more time visiting flowers and collecting more pollen grains. 

*H. mutabilis* compensated for the deficiency in the special pollinators (moth pollinators) in the introduced habitats by attracting more bee pollinators to provide pollination services. There are differences between moth and bee pollinators in the influence of pollinator size on pollination efficiency due to the differences in their body parts in contact with the two sexual organs of *H. mutabilis* flowers. There was a significant positive correlation between the size of bee pollinators and pollination efficiency, while there was a significant positive correlation between the length of the proboscis of *M. pyrrhosticta*. However, *H. mutabilis* is widely cultivated as an ornamental garden plant, and its flowering period lasts a very long time. Are there any differences in the reproductive strategies between the different flowering periods? Are there any differences in the reproductive strategies of *H. mutabilis* in different introduced habitats? Next, we will study the reproductive strategy of *H. mutabilis* further.

## Figures and Tables

**Table 1:** Floral traits of *Hibiscus mutabilis* (number of flowers: *n* = 30).

Floral Traits	Mean ± SE
Diameter of corolla (mm)	128.79 ± 1.54
Height of corolla (mm)	30.97 ± 1.15
Length of sepal (mm)	29.52 ± 0.53
Width of sepal (mm)	13.80 ± 0.30
Length of petal (mm)	68.57 ± 0.93
Width of petal (mm)	57.12 ± 0.68
Length of stamen (mm)	6.85 ± 0.04
Length of staminal column (mm)	35.99 ± 0.50
Pistil length (mm)	61.02 ± 0.82
Height of ovary (mm)	9.19 ± 0.17
Diameter of ovary (mm)	12.36 ± 0.07
Circumference of floral base (mm)	25.70 ± 0.30
Number of stamens per flower	141.77 ± 2.30
Number of pollen per flower	72,813.30 ± 1220.51
Number of ovules per flower	241.93 ± 2.80
Pollen/ovule ratio	303.22 ± 5.91
Nectar volume (μL)	24.61 ± 1.08
Sugar concentration (g/100 mL)	29.33 ± 1.22

## Data Availability

All data are included in https://doi.org/10.5061/dryad.qrfj6q5pk, and further inquiries can be directed to the corresponding author.
